# Impact of home healthcare on end-of-life outcomes for people with dementia: a systematic review

**DOI:** 10.1186/s12877-022-02768-3

**Published:** 2022-01-27

**Authors:** Ping-Jen Chen, Lisanne Smits, Rose Miranda, Jung-Yu Liao, Irene Petersen, Lieve Van den Block, Elizabeth L. Sampson

**Affiliations:** 1 Marie Curie Palliative Care Research Department, Division of Psychiatry, University College London, Maple House, 149 Tottenham Court Rd, Bloomsbury, London, W1T 7BN UK; 2 Department of Family Medicine and Division of Geriatrics and Gerontology, Kaohsiung Medical University Hospital, Kaohsiung, Taiwan; 3 School of Medicine, Kaohsiung Medical University, Kaohsiung, Taiwan; 4 Faculty of Medicine, University of Amsterdam, Amsterdam, the Netherlands; 5 End-of-Life Care Research Group, Vrije Universiteit Brussel (VUB) and Ghent University, Brussels, Belgium; 6 Department of Family Medicine and Chronic Care, Vrije Universiteit Brussel (VUB), Brussels, Belgium; 7 Department of Public Health, Kaohsiung Medical University, Kaohsiung, Taiwan; 8 Research Department of Primary Care and Population Health, University College London, London, UK; 9 Barnet Enfield and Haringey Mental Health Trust Liaison Psychiatry Team, North Middlesex University Hospital, London, UK

**Keywords:** Home healthcare, Palliative care, Acute healthcare utilization, Advance care planning, Dementia, End-of-life

## Abstract

**Background:**

Home healthcare (HHC) comprises clinical services provided by medical professionals for people living at home with various levels of care needs and health conditions. HHC may reduce care transitions from home to acute hospitals, but its long-term impact on homebound people living with dementia (PLWD) towards end-of-life remains unclear. We aim to describe the impact of HHC on acute healthcare utilization and end-of-life outcomes in PLWD.

**Methods:**

Design: Systematic review of quantitative and qualitative original studies which examine the association between HHC and targeted outcomes. Interventions: HHC. Participants: At least 80% of study participants had dementia and lived at home. Measurements: Primary outcome was acute healthcare utilization in the last year of life. Secondary outcomes included hospice palliative care, advance care planning, continuity of care, and place of death. We briefly reviewed selected national policy to provide contextual information regarding these outcomes.

**Results:**

From 6831 articles initially identified, we included five studies comprising data on 4493 participants from USA, Japan, and Italy. No included studies received a “high” quality rating. We synthesised core properties related to HHC at three implementational levels. Micro-level: HHC may be associated with a lower risk of acute healthcare utilization in the early period (e.g., last 90 days before death) and a higher risk in the late period (e.g. last 15 days) of the disease trajectory toward end-of-life in PLWD. HHC may increase palliative care referrals. Advance care planning was an important factor influencing end-of-life outcomes. Meso-level: challenges for HHC providers in medical decision-making and initiating palliative care for PLWD at the end-of-life may require further training and external support. Coordination between HHC and social care is highlighted but not well examined. Macro-level: reforms of national policy or financial schemes are found in some countries but the effects are not clearly understood.

**Conclusions:**

This review highlights the dearth of dementia-specific research regarding the impact of HHC on end-of-life outcomes. Effects of advance care planning during HHC, the integration between health and social care, and coordination between primary HHC and specialist geriatric/ palliative care services require further investigation.

**Supplementary Information:**

The online version contains supplementary material available at 10.1186/s12877-022-02768-3.

## Introduction

Dementia is a life-limiting, progressive neurodegenerative syndrome affecting multiple cognitive and physical functions [1]. It is currently one of the most common causes of death in high-income countries, and globally, leads to an escalating need for end-of-life care [2–4]. People living with dementia (PLWD) are at a high risk of experiencing care transitions (i.e. transfer from home or care homes to acute hospital admission or emergency department visit), particularly towards the end-of-life [5–8]. Research in the USA and Taiwan has shown that potentially non-beneficial life-sustaining treatments such as tube feeding or mechanical ventilation are associated with care transitions [9–11] which may not improve the quality or length of life and are burdensome for the PLWD and their carers [7, 8, 10, 12]. Strategies to support this population with complex care needs and high care cost living and dying well in the community are vital [13, 14].

Palliative care has been considered an important service that contributes to the quality of care and fulfills the care needs of PLWD near the end-of-life [15]. According to World Health Organization’s definition, palliative care is an approach that involves the identification and management of problems associated with a life-threatening illness for patients and their families, prevents or relieves their suffering and improves their quality of life [16]. Its impact on reducing transitions for PLWD in care homes has been investigated [17–19]; however, the evidence on effective palliative care for PLWD living at home remains scarce and inconclusive [20, 21]. Furthermore, the provision of palliative care for PLWD is at a low coverage level across countries and the referral of the service is usually late in the potentially long, slow decline of disease trajectory when PLWD are approaching their end-of-life [11, 22, 23]. It is important to better understand the long-term impact on interventions such as home healthcare (HHC), which is usually provided earlier and more common for PLWD living at home, on reducing their high risk of acute healthcare utilisation or other health outcomes towards the end-of-life [15, 24, 25].

HHC comprises a spectrum of clinical care provided by healthcare professionals for people living at home with various levels of care needs and health conditions at different stages throughout the life course [26]. HHC types vary in terms of acuity, type of care provided, and degree of physician involvement, including patient-centered medical home, hospital at home, home-based primary care, physician or nurses house calls, skilled home healthcare, rehabilitation, and medication managements [26, 27]. HHC does not include case management, exercise coaching, social care (such as hygiene care or nutrition support) or self-management.

HHC is increasingly recognised as an integrated and value-based service for the ageing population including PLWD [14]. The demand for promoting better integrated and continuous care at home has been advocated by groups of PLWD, caregivers, and health professionals [27–29], and some reforms of policy to integrate the fragmented services and quality-based payment schemes to enhance multidisciplinary approach at a national level for improving quality of HHC is established in high-income countries [27, 29–33]. However, in the existing literature of HHC, only a few studies have focused on the PLWD and none of them reviewed long-term effects of HHC on acute healthcare utilisation and outcomes at the end-of-life [34, 35].

### Aims

1. To investigate the effects of primary HHC on the acute healthcare utilisation at the end-of-life among PLWD, including hospital, emergency department, intensive care, aggressive procedures, medications, and care transitions.
2. To understand the association between primary HHC and use of hospice palliative care, continuity of care, and place of death among PLWD.
3. To identify the policy or regulations that may influence the impact of HHC on the aforementioned outcomes among PLWD.

## Methods

We registered the protocol on PROSPERO (CRD42019151250)and adhered to PRISMA statement in reporting the review [36].

### Eligibility criteria

This review included peer-reviewed original articles of quantitative and qualitative studies. The inclusion and exclusion criteria on the study design, definition of PLWD and HHC and details of the study outcomes are summarised in Table 1. The comparison groups include any type of usual care, routine care or no intervention. In this study, home-based palliative care is not included in the HHC interventions because the effects of the services for PLWD have been reviewed [21], and palliative care is identified as an outcome of interest.

**Table 1:** Inclusion criteria for eligible studies

Population	Intervention	Outcome
· At least 80% of study participants had a clinical diagnosis of dementia and lived at home · Data of people with dementia (if < 80% of study participants) were analysed separately	***Primary home healthcare*** · Provided by health care professionals · At least include physicians or nurses · Examples - Home-based primary care - Skilled home health care - Patient-centered medical home - Physician or nurses house calls - Hospital at home - Medication management - Rehabilitation · Exclude - Home-based palliative care - Routine dialysis or respiratory care - Hygiene care - Nutrition consultation - Exercise coaching - Other social care services - Self-management - Case management	Primary · ***Acute healthcare utilization in the last year of life*** - Hospitalization or intensive care unit admission - Length of hospital or intensive care unit stay - Emergency department visits - Transition of care - Life-sustaining treatments - Aggressive procedures - Drug prescriptions Secondary · ‘Continuity of care’ in the last year of life · Use of hospice palliative care including advance care planning at any time after the start of home healthcare · Place of death
**Study design**		
· Any type of trials · Uncontrolled before and after studies · Interrupted time series · Observational studies · Qualitative study · Exclude Reviews, case reports, commentaries, conference abstracts, qualification theses, and non-English articles		

### Search strategy and study selection

We applied a three-step search strategy: An initial limited search of Medline was performed, followed by the analysis of the terms used in titles and abstracts, and of the index terms used to describe articles. A second search using all identified keywords and index terms for ‘dementia’, ‘home healthcare’, and a series of outcomes such as ‘acute healthcare utilisation’, ‘continuity of care’, ‘palliative care’ or ‘place of death’ were then undertaken across five electronic databases, including OVID Medline, EMBASE, PsycINFO, Cochrane Library and CINAHL, from inception to September 2020. In the third search, the reference list of all identified articles was searched for additional studies. Search terms were used in combination with MESH headings, controlled vocabulary and free-text terms to cover the topics and detail (see Supplementary file).

Two authors (PJC and LS) read the abstracts of half of the retrieved records to identify potentially relevant publications. These publications were marked as ‘include’ or ‘uncertain’ after the exclusion of irrelevant studies. A random 15% of selected records were independently checked by a second reviewer (JYL). The two authors then retrieved the full texts of identified studies and screened them according to the eligibility criteria. The final list of articles was checked by the three authors and any disagreements were discussed with the third reviewer (ELS) to reach consensus. We constructed a PRISMA flowchart to describe the selection process and a table containing excluded studies with the rationale for exclusion. References were managed and deduplicated by citation management software.

### Data extraction

We extracted relevant data into a standardised table using Microsoft Excel. The table format was pilot-tested on three articles to ensure consistency and was approved by the research team. Extracted data included country, time, study design, data source and collection, research questions (aims), participants, content of interventions, comparison, and outcomes. Information from included studies was extracted by PJC and JYL independently and checked for accuracy by RM. Discrepancies were discussed with ELS to reach consensus.

### Quality assessment

The Critical Appraisal Skills Programme (CASP) toolkit was used by PJC and LS to appraise the quality of the included studies [37]. Studies were rated as strong, moderate or weak based on the following components: study design, data collection method, bias of selection and outcome measurements, intervention integrity, confounding factors, appropriate analysis and implication for practice. Discrepancies were discussed with RM to reach a consensus.

### Data synthesis

We narratively described the effectiveness of HHC-related outcomes. We used an adapted multilevel framework of Ferlie and Shortell [38] to synthesise core properties of HHC. The empirically derived model was used for summarising and classifying the various characteristics related to end-of-life care provision in care homes across countries and focus on three levels of the implementation: macro- such as national policy, legislation, or financial provision; meso- such as training or service model/framework; micro- effects or components in an individual programme [39]. We were unable to conduct a meta-analysis because of clinical and statistical heterogeneity across studies. Results were set out in a table with data reported from the included study (e.g. *p*-values).

## Results

**Fig. 1:**
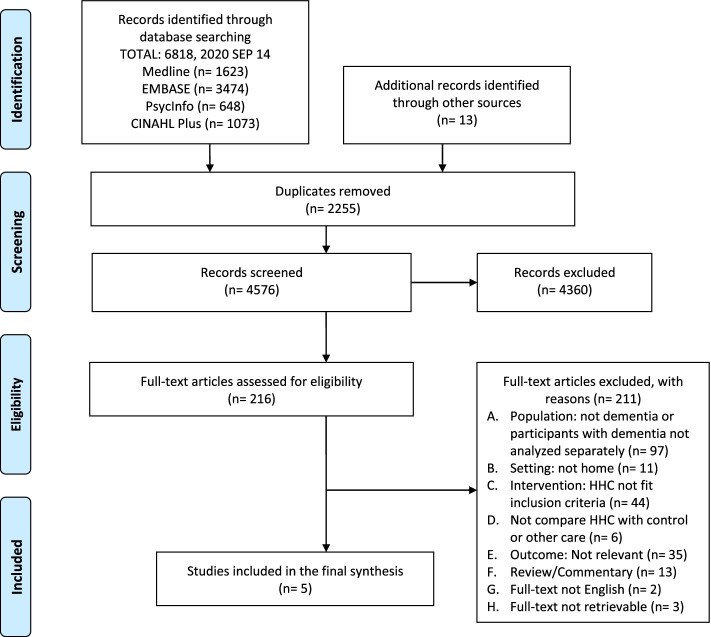
The Preferred Reporting Items for Systematic Reviews and Meta analyses (PRISMA) flowchart of the selection process

Of all retrieved studies, five met the inclusion criteria. The selection process is illustrated in the PRISMA flow chart (Fig. 1).

### Studies’ characteristics

We identified five studies; one prospective cohort study [40], two retrospective cohort studies [41, 42], and two case-control studies (Table 2) [43, 44]. No clinical trials or qualitative studies were included. In total, 4493 participants with dementia were included in these quantitative papers. Three studies (3936 participants, 87.6%) were conducted in the USA [41, 42, 44], and one each from Italy [40] and Japan [43]. All studies included both males and females and most participants were over 80 years old. The HHC provided in these five studies were all forms of home-based primary care.

**Table 2:** Characteristics of five studies included in the review and information regarding home healthcare for people with dementia

Study	Design	Aim	Data source	Participants	HHC intervention	Comparison	Quality
**Arai** **2020** [43] Japan	Observational, retrospective case-control. Quantitative.	To examine and compare the outcomes of fever treatment between home care patients who received home treatment and those who were hospitalized for treatment.	Medical records from Iki-iki Clinic, Japan. 1st April 2007 to 31st March 2017.	61 people aged ≥65 develop a fever of ≥38.0 C during home care. (98% Degree of Independent Living for the Elderly with Dementia ≥1); 40 in the HHC group, 21 in the hospitalized group.	Home care on a 24-h, 365 days a year basis was provided by doctors’ and nurses’ visits regularly. The treatment for the fever episode was kept at home.	Transfer and admission to nearby hospitals after the fever episode.	Weak
**Jennings 2019** [41] United States of America	Observational, retrospective cohort. Quantitative.	To examine the impact of a coordinated care program that specifically focused advance care planning on end-of-life care in homebound people with dementia	Epic-based electronic health records in the University of California, Los Angeles and the other two hospital systems in West Los Angeles. 1st July 2012 to 1st July 2016.	332 people with dementia; 184 in HHC with POLST group, 138 in HHC without POLST group.	Alzheimer’s and Dementia Care program, a longitudinal comprehensive nurse practitioner dementia management program, with the completion of POLST. POLST, a document on physician orders for a series of life-sustaining treatments that a patient wishes to receive or refuse towards the end-of-life.	Alzheimer’s and Dementia Care program without the completion of POLST	Weak
**Toscani 2015** [40] Italy	Observational, multicenter prospective cohort. Quantitative.	To describe and compare critical decisions made by health care professionals for people with dementia in nursing homes and home care.	The End-Of-Life Observatory: Prospective Study on DEmentia patients Care study. June 2007 to May 2009.	496 people with advanced dementia (Functional Assessment Staging Tool≥7); 181 in the HHC group, 315 in the nursing home group.	Multidisciplinary team consists of visiting nurses, general practitioners who provide disabled older people with assistance at home in the Emila Romagna area. HHC may also include visits by psycho-geriatricians, palliative care consultants, social workers, and volunteers.	Nursing home staff comprise physicians, nurses, physiotherapists, psychologists, and health aides. Nursing homes differ widely in the numbers of inpatients and services offered (ie, occupational therapy, dementia day care, cognitive therapy, music therapy, etc.).	Moderate
**Wilson 2015** [44] United States of America	Observational, retrospective case-control (propensity score matching). Quantitative.	To describe the differences in health care utilization and costs between elder people who received home-based care and the control group.	Medicare utilization records from JEN data management and consulting associates. 2004 to 2006.	584 people with dementia; 144 in the House Calls program, 440 in the control group.	The House Calls program - Developed to provide medical care to frail older people, including those with dementia - Focused on continuity of care, integrated care based on patients’ needs, coordinated team-based approaches. - Delivered by a team of physicians, nurse practitioners, non-clinical care managers, and social workers.	Patients enrolled in Medicare but not participating in the House Calls program.	Weak
**Mitchell 2004** [42] United States of America	Observational, retrospective cohort. Quantitative.	To examine and compare the end-of-life experience of people with severe dementia who died within one year of admission to a nursing home or a home care service.	State of Michigan (1) Minimum Data Set -Nursing Home Version 2.0 (*n* = 121,129 people in 458 facilities). 1st July 1998 to 31st December 2000. (2) Minimum Data Set -Home Care (*n* = 23,095 people). 1st October 1998 to 31st December 2001.	3020 people aged ≥65 with advanced dementia (Cognitive Performance Scale =5/6); 290 in the HHC group, 2730 in the nursing home group.	Michigan Choice Waiver for the Elderly and Disabled program. The waiver program provides a wide range of home care agency-based services, including nursing care, personal emergency response systems, and other social care support.	Nursing home care in all facilities in Michigan	Weak

*HHC* home healthcare, *POLST* physician orders for life-sustaining treatment

#### Micro-level

Four studies mentioned that the HHC was provided by the multidisciplinary team [40, 42–44]. HHC in the other study emphasised advance care planning in a nurse-led programme [41]. Only one study mentioned the duration of the HHC intervention [42], and no study reported the time between dementia diagnosis and the first HHC.

Two studies compared outcomes in people receiving HHC with those in nursing home care [40, 42], and another study compared outcomes of treatments for acute events between the HHC group and the hospitalised group [43]. Four studies investigated outcomes related to end-of-life issues or palliative care [40–42, 44], whereas survival and mortality rate was the main outcome outcomes in the other study [43].

#### Meso-level

Three studies mentioned coordination with social workers, non-clinical social care support and external specialists such as palliative care consultants or geriatricians in HHC [40, 42, 44].

#### Macro-level

We did not find information on national policy or financial schemes that influence HHC for PLWD in the selected articles.

### Quality of the evidence

No study received a high-quality rating (Table 2). The main reasons for low scores were authors not taking account of confounding factors appropriately in the analysis and the absence or insufficiency of follow-up period because of study design.

### Impact of HHC on end-of-life outcomes for PLWD

Results identified from included papers were summarised in Table 3. All the observed impacts were at the micro-level.

**Table 3:** Effects of home healthcare on end-of-life outcomes in people with dementia

**A. Primary outcome measure**		
**Acute healthcare utilization in the last year of life**		
Author, year	Outcome of interest	Results
Toscani 2015 [40]	**Physicians feel it difficult to decide for patients’ hospitalization** (when patients’ prognosis of survival <= 15 days)	**HHC group vs. Nursing home care group** **25.5% vs. 3.1%,** ***p <*** **0.001**
Mitchell 2004 [42]	**Hospitalization within 90 days prior to the last** Minimum Data Set **assessment**	**HHC group vs. Nursing home care group** **31.5% vs. 43.7%,** ***p <*** **0.001**
	**Emergency department visit within 90 days prior to their last** Minimum Data Set **assessment**	13.1% vs. 11.4%, *p* = 0.41
	**Procedures use**	
	1. Oxygen therapy within 14 days prior to their last Minimum Data Set assessment	**12.5% vs. 24.4%,** ***p <*** **0.001**
	2. Feeding tube (time frame not mentioned)	**11.9% vs. 27.2%,** ***p <*** **0.001**
	3. Intravenous therapy	2.8% vs. 3.6%, *p* = 0.52
	4. Foley catheter	**15.8% vs. 29.4%,** ***p <*** **0.001**
	**Medication use within 7 days prior to their last** Minimum Data Set **assessment**	
	1. Antipsychotic	19.7% vs. 22.7%, *p* = 0.35
	2. Antianxiety	**20.7% vs. 15.9%,** ***p*** **= 0.05**
	3. Antidepressant	24.7% vs. 21.5%, *p* = 0.21
Jennings 2019 [41]	**Hospitalization in the last 6 months** 1. Any hospitalization	**HHC with POLST vs. HHC without POLST group** **43% vs. 31%,** ***p*** **= 0.04**
	2. > 1 Hospitalization	**22% vs. 12%,** ***p*** **= 0.02**
	3. Length of stay in hospital, median (interquartile range)	5.8 (3.7–8.3) days vs. 4.1 (3.1–8.9) days, *p* = 0.22
	**Emergency department visit in the last 6 months**	
	1. Any emergency department visit	29% vs. 23%, *p* = 0.27
	**Intensive care unit admission in the last 6 months**	
	1. Any intensive care unit admission	6% vs. 4%, *p* = 0.62
	2. Length of stay in intensive care unit, median (interquartile range)	2.0 (1.0–3.4) days vs. 5.8 (0.4–11.7) days, *p* = 0.41
**B. Secondary outcome measures**		
**Hospice and palliative care use**		
Author, publication year	Outcome of interest	Results
Toscani 2015 [40]	**Physicians feel it difficult to decide for deep or terminal sedation**	**HHC group vs. Nursing home care group** 0.7% vs. 1.41%, *p* = 0.49
	**Purpose of all the decisions made for**	
	Reduce symptoms/suffering	57% vs. 81.1%
	Avoid/stop futile treatments	10.3% vs. 8%
	Improving the quality of death by minimizing suffering	0% vs. 1.6%
Mitchell 2004 [42]	**Hospice care referral any time prior to death**	**HHC group vs. Nursing home care group** **13.1% vs. 5.7%,** ***p <*** **0.001**
Jennings 2019 [41]	**Hospice care discussion or consultation in the last 6 months of life**	**HHC with POLST vs. HHC without POLST group** **78% vs. 64%,** ***p*** **= 0.01**
	**Died in hospice care**	**74% vs. 62%,** ***p*** **= 0.03**
Wilson 2015 [44]	**Hospice use**	**House Calls group vs. Control group** **22.9% vs. 8.9%,** ***p <*** **0.05**
**Advance care planning**		
Author, publication year	Outcome of interest	Results
Mitchell 2004 [42]	**Advance directive** any time prior to death	**HHC group vs. Nursing home care group** **39.4% vs. 57.4%,** ***p*** **< 0.001**
**Place of death**		
Author, publication year	Outcome of interest	Results
Jennings 2019 [41]	**Home death**	**HHC with POLST vs. HHC without POLST group** **70% vs. 59%,** ***p*** **= 0.04**
Arai 2020 [43]	**Place of death** (within 90 days after fever onset)	**HHC group vs. Hospitalized group** 12.5% died (at home) vs. 33.3% died (in hospital)

*HHC* home healthcare, *POLST* Physician Orders for Life-Sustaining Treatment

#### Acute healthcare utilisation in the last year of life

Three studies have reported results regarding our primary outcomes of interest [40–42]. In the Italian cohort study, a higher percentage of HHC physicians felt it difficult to decide whether PLWD should be hospitalised or not than physicians practising in nursing homes (25.5% vs. 3.1%, *p* < 0.001) when patients’ estimated survival was fewer than 15 days [40]. In the USA, however, Mitchell et al. reported that fewer people with advanced dementia receiving HHC were hospitalised within 90 days before the last Minimum Data Set assessment compared with those cared for in nursing homes (31.5% vs. 43.7%, *p <* 0.001) during the period from 1998 to 2001 [42]. In terms of specific procedures, fewer people in the HHC group were given life-supporting therapies such as oxygenation or feeding tube than those in the nursing home group at the end-of-life.

Jennings et al. described the effects of an HHC programme in California, which specifically focused on advance care planning including Physician Orders for Life-Sustaining Treatment (POLST), on end-of-life care in PLWD [41]. A higher proportion of PLWD who received HHC with the completion of POLST experienced hospitalisations in the last 6 months of life compared with those receiving HHC but without a POLST. No significant difference was found between the length of hospital stay, intensive care unit admission, and frequency of emergency department visits between the two groups.

#### Hospice palliative care use

In Toscani’s study, physicians in the nursing home group were more likely to consider/make decisions that focused on reducing suffering or on improving quality of death for PLWD than physicians providing HHC [40]. Two studies in the USA reported a higher percentage of HHC recipients who used hospice or were referred to hospice care before their death compared with nursing home residents or the control group [42, 44]. In California, Jennings et al. demonstrated that PLWD who received HHC with a completed POLST were more likely to have hospice care discussion or consultations, use hospice care when they died and died at home than HHC recipients who did not have a completed POLST [41].

#### Advance care planning

Only Mitchell’s study indicated that fewer HHC recipients had advance directives before death than did nursing home residents, despite a higher proportion of HHC recipients having a life expectancy of less than 6 months [42].

#### Place of death

Each study in the USA and Japan reported this outcome [41, 43]. A higher proportion of PLWD in HHC with POLST died at home than those in HHC without POLST. Information regarding PLWD’s preferences of place of care/ death is not found.

## Discussion

To the best of our knowledge, this is the first review that explored the association between primary HHC and end-of-life outcomes among homebound PLWD who are at a high risk of mortality [24, 42]. The comparison groups and outcomes measured in the five included studies vary, and we found the results were heterogeneous and very limited to conclusively examine the effects of HHC on end-of-life outcomes. The existing literature suggests that HHC may be associated with an inverse risk of acute healthcare utilisation in the early and late periods (e.g. 90 vs 15 days before death) of the disease trajectory towards the end-of-life in PLWD. HHC seems to increase referrals to hospice palliative care, whilst advance care planning may influence the effects of HHC on end-of-life outcomes. HHC providers’ difficulty in making treatment decisions for PLWD at the end-of-life may require further training and external support. The coordination between HHC and social care is important but not well implemented and investigated.

### Micro-level

The differential effects of HHC on acute healthcare utilisation among PLWD in the early or late period imply different care needs at various stages in the disease trajectory among PLWD, for which distinct components and models of HHC service may meet their needs better. A systematic review showed that home-based primary care mostly reduces the events and length of hospitalisation [35]; however, this effect was observed within 1 year after HHC but not followed up to the recipients’ death. In addition, we have not been able to clarify the influence of the duration, continuity, or intensity of HHC from the current literature. Among homebound PLWD approaching the end-of-life, identifying care needs and treatment decisions are complicated because the person may be unable to express their care preferences [40]. Multidisciplinary approaches in HHC may be beneficial to PLWD towards the end-of-life [40, 42–44], but none of the selected studies have examined the effectiveness of skill mix across various professions or quantified the contribution of each discipline.

Given that people with dementia may lose the capacity to make decisions, advance care planning in HHC may substantially influence end-of-life outcomes. A recent review showed that advance care planning for PLWD was associated with decreased hospitalisations and increased concordance between prior preferences and actual care received [45]. In primary care settings, key barriers of professionals to conduct advance care planning for PLWD includes the time restraints of medical staffs; the insufficiently trusted relationship between PLWD and medical staffs due to infrequent contact; staffs’ attitude, knowledge, skills, and moral considerations towards talking end-of-life issues and death; and inadequate reimbursement [46, 47]. However, the context of home visits is preferred by PLWD and family caregivers because it is a trusted environment to have care plan conversations addressing not only medical but also non-medical preferences, such as valued abilities and activities, family support and relationship, and place of care/ death [46, 48]. Both interactive training and clinical practice of conducting advance care planning during HHC can be facilitated by involving interdisciplinary professionals, such as nurses, social workers, or care managers [46, 48].

### Meso-level

Providing training programmes and seamless palliative care support from external specialists to HHC practitioners may improve the capability of primary end-of-life care in the home setting [49, 50]. HHC physicians were less likely to initiate palliative care for PLWD [40], even though eventually the specialist palliative care or hospice referral is higher for people in the HHC group than those in the nursing home or control group [42, 44]. Primary healthcare workers may more likely to consider that palliative care is not meaningful in PLWD than in people with other life-limiting illnesses [51] or only acknowledged its benefit in terminal care, so they refer the people to the service late [49]. The resistance of timely palliative care approaches provided by HHC professionals, such as symptom management and initiating advance care planning discussion, can be further overcome through education, skills training, and discussion of moral dilemmas [47, 49, 52]. Further service commissioning and integration between HHC teams and external specialists such as geriatricians or palliative care may contribute to PLWD living and dying well at home [23].

Good-quality HHC requires strong coordination between health and social care services to achieve better end-of-life outcomes. A UK cohort study found that the need for social care services increased among PLWD towards the end-of-life [23], and the lack of social care support at home may lead to a higher risk of acute healthcare use [53]. In the selected studies, only one US study assessed the use of social services in the HHC setting and found such services were not used to its full potential, showing that the coordination between health and social care may be an area for improvement [44]. The barriers of care integration for PLWD towards the end-of-life may include the conflicting relationships and communication between disciplines and settings, lengthy referral processes, minimal care planning, diffuse responsibility, and fragmented reimbursement system [50, 54]. At the local services, organising the cross disciplinary network between healthcare and social care sectors, increasing the communication and establishing the practice guidelines with shared goals, peer supporting to formal and family caregivers, and highlighting good practices can improve the quality of integrated care [50].

### Macro-level

Reform of national policy and payment schemes would be vital in promoting better care for individuals [55, 56], or in building up interdisciplinary collaborations and delivery systems between health and social care services [50]. However, contextual information, including descriptions of the related policy and payment schemes in each country, was not mentioned in the selected papers.

To understand this context better, we summarise some international examples through a brief policy review and discussion in our research network (Table 4), including those from where the five papers of this review originated (USA, Italy and Japan) and the top-ranked countries in related regions in the Quality of Death Index Report [56], such as Australia, Canada, UK, and Taiwan. The key lessons from the policy comparison are building up person-centred continuous care at home, with a seamless connection between primary care and palliative care throughout the disease trajectory, quality- and value-based payment for interdisciplinary collaboration, as well as comprehensive networking with coordination between health and social care sectors [26–28, 31].

**Table 4:** International comparison of the policy and payment scheme that support better home healthcare for the people with dementia

Country^a^	National policy	Payment scheme or financial resource	Descriptions
**USA** Ranked 1st in America in the QODI Report [56]	Accountable Care Organizations (2010) [27], Bundled Payments for Care Improvement (2013) [30]	National health insurance (Medicare, Medicaid)	Switch fee-for-service payments to quality- and value-based purchasing program that promotes home health use and the coordination of home-based social care services
**Japan** Ranked 3rd in Asia in the QODI Report	Community-based Integrated Care System (2012) [28, 32]	Mandatory health and long-term care insurance; and social security system	Improve coordination between medical care and welfare services at home or in the community. Incentives for both health and long-term care insurance have been increased and integrated for encouraging care managers’ coordination of early discharge support, physicians who advise care managers in home-based care, and home-based medical care
**Australia** Ranked 1st in Pacific in the QODI Report	Health Care Homes^b^ (2016) [29]	Pilot model with bundled payment from government fund	Improve access to, and continuity of, integrated and personalised care at home; new funding models that allow employment of re-conceived roles, such as care coordinators or social workers; enhance coordination with local health networks on the basis of better communication and shared goals
	Home Care Package (2013) [57], Commonwealth Home Support Programme (2016) [58]	General taxation	Care need assessed by Aged Care Assessment Team or the Regional Assessment Service; support the wound care, general health consultation and education; coordinate with the medical team for treatments needed
**Italy** Ranked 13th in Europe in the QODI Report	Dementia National Plan (2014) [59]	National Insurance and general taxation	Improve the quality of care delivered at home by promoting the training of health- and social care professionals and developing shared activities involving general practitioners and carers
**Taiwan** Ranked 1st in Asia in the QODI Report	Integrated Home Care Project (2016) [31]	Mandatory national health insurance	Universal payment scheme aims to enhance the continuity and integration of series of home-based healthcare. Multidisciplinary team services including dentists, traditional Chinese medicine physicians, psychologists are reimbursed
	Long-term Care Plan 2.0 (2016) [60]	Taxes on inheritance, tobacco, gift, and house or land transactions income.	Allocate more resources on community and home-based social care in coordination with home-based medical care
**Canada** Ranked 2nd in America in the QODI Report	Principles on Shared Health Priorities (2017) [33]	National Insurance and general taxation	Spread and scale integrated models of home care; enhance access to palliative and end of life care at home; increase support for caregivers; improve home care infrastructure, such as digital connectivity, remote monitoring technology
**UK** Ranked 1st in Europe in the QODI Report	The NHS Long Term Plan, NHS England (2019) [61]	National Insurance and general taxation	For patients to receive more options, better support, and properly joined-up care at the right time in the optimal care setting
	Better Care Fund: Policy Framework (2019–20) [62]		NHS England will provide “Comprehensive Model for Personalised Care” for up to 2.5 million people by 2023/24. Promotes funding of care in place of person’s choice

^a^Country is sorted by the year of policy formulation. *QODI* Quality of Death Index. ^b^Program ended on 30th June 2021

### Strengths and limitations

We comprehensively and systematically searched the literature by applying a wide range of search terms including synonyms of HHC and types of HHC programmes. The identified studies were rigorously checked by quality assessment tools. The international members of our research team provided insights and interpretation.

This study has several limitations. Firstly, the majority of studies evaluating the effects of HHC had short follow-up periods, often less than 12 months after the initiation of HHC [34, 63, 64]. Data about end-of-life outcomes that occurred within the final years before death were neither investigated nor analysed separately, leading to fewer papers meeting our selection criteria [64, 65]. Secondly, none of the included studies were rated as high quality in critical appraisal. Outcomes were heterogeneous, and it was not possible to pool data and perform a meta-analysis of the findings. In addition, the lack of information about the duration, intensity or components of HHC interventions meant that we could not explore the ‘dose-response’ relationship between characteristics of HHC and outcomes [66]. Finally, the small sample size in the HHC group and lack of random sample selection may lead to poor external validity [67].

### Implications for research and practice

The sparse evidence in our review suggests that the role of primary HHC may have been overlooked as a key service that could deliver better quality end-of-life care for PLWD. HHC services may vary widely across countries, and details of the components and contextual factors of HHC and how they are implemented are important to evaluate the effects of complex interventions, which were not reported in the included studies [68]. For future studies, it is essential to better understand the effective components of HHC, such as advance care planning, and the mechanism of how they influence end-of-life outcomes for PLWD.

Conducting a randomised trial or a prospective cohort study with a longer follow-up period of HHC would be challenging in practice [24, 35]. A more pragmatic, hybrid paradigm incorporating quality improvement or service evaluation may be more useful and realistic to conduct [69]. Large real-world datasets containing whole population samples, with complete follow-up are also good sources to evaluate HHC programmes throughout the disease trajectory, though appropriate and robust methodologies should be applied [67, 70–72]. This would reduce selection bias and prevent missing data due to the attenuation of study cohorts. Current metrics of care quality, which were developed for individual diseases, are not holistic and do not capture more value-based dimensions such as continuity of care or level of care integration [73].

Regarding the clinical practice, advance care planning, comprehensive geriatric assessment, and a palliative approach which focuses on patients’ care preferences and improving quality of life should be emphasised in HHC for PLWD [15, 74, 75]. Stakeholders should enhance education for HHC users and providers, strengthen the training of the interdisciplinary workforce, and promote a service model supported by external professionals including social care or even telemedicine during the pandemic to meet complex end-of-life care needs in PLWD [23, 26, 49, 74]. Policymakers are encouraged by the experience of national policy and payment scheme reform in some countries to build up the continuously integrated care framework that improves the synergy of various services [26, 27, 74].

## Conclusion

This review added the new knowledge that different care needs at various stages in the disease trajectory towards the end-of-life among PLWD urge more integrated services with effective components to respond to their demand better. Effects of advance care planning, multidisciplinary approach, integration between health and social care, and coordination between primary HHC and specialists’ support in local healthcare networks for better continuity of care at home should be emphasised in clinical practice and policy-making. Population-based large databases may provide opportunities to examine more clearly the long-term impact of HHC and its synergy with other clinical services on end-of-life outcomes in a longitudinal study design.

## Supplementary Information

**Additional file 1: Supplementary file.** Search strategy and terms in electronic databases.

## Data Availability

All data generated or analyzed during this study are included in this published article and its supplementary files.
