# Supplementary material for: Impact of home healthcare on end-of-life outcomes for people with dementia: a systematic review
Source: BMC Geriatr. 2022 Jan 27;22:80. doi: 10.1186/s12877-022-02768-3 (PMC8793202; doi:10.1186/s12877-022-02768-3)
Supplement: Supplementary file 1 — Additional file 1: Supplementary file. Search strategy and terms in electronic databases. [file 12877_2022_2768_MOESM1_ESM.docx]

| **Search strategy and terms in electronic databases** | |
| --- | --- |
| **No.** | **Search strategy** |
| 1 | exp dementia/ |
| 2 | dementia.tw. |
| 3 | Alzheimer.tw. |
| 4 | 1 or 2 or 3 |
| 5 | exp Home Care Services/ |
| 6 | (hospital adj2 home).tw. |
| 7 | home hospitalization.tw. |
| 8 | ((home or home-based) adj (primary care or medical care or health care or healthcare)).tw. |
| 9 | (patient-centered medical home or patient centered medical home or PCMH).tw. |
| 10 | exp Primary health care/ |
| 11 | exp General practice/ |
| 12 | ((home* or domiciliary or primary or community* or outreach* or health* or medical or integrate* or mix*) adj2 (service or care or treatment or therapy or intervention or nursing or visit)).ti. |
| 13 | 10 or 11 or 12 |
| 14 | (home* or house* or housing or community*).ti. |
| 15 | 13 and 14 |
| 16 | 5 or 6 or 7 or 8 or 9 or 15 |
| 17 | (hospital* or admission or readmission or inpatient*).tw. |
| 18 | ((emergency or casualty) adj (room or department or service or ward)).tw. |
| 19 | (accident and emergency).tw. |
| 20 | exp Life Support Care/ |
| 21 | (life-saving or life-sustaining or resuscitat*).tw. |
| 22 | ((health* or medic* or treat* or interve* or drug or prescrib* or surgery or procedur* or therap*) adj2 (utilization or utilisation or usage)).tw. |
| 23 | 17 or 18 or 19 or 20 or 21 or 22 |
| 24 | exp patient transfer/ |
| 25 | ((continu* or constan*) adj2 (care or service)).tw. |
| 26 | ((continu* or constan*) and (care or service)).tw. |
| 27 | (transition* and (care or service)).tw. |
| 28 | 24 or 25 or 26 or 27 |
| 29 | exp Palliative Care/ |
| 30 | exp Palliative Medicine/ |
| 31 | exp Terminal Care/ |
| 32 | exp Hospice Care/ |
| 33 | exp Hospices/ |
| 34 | supportive care.tw. |
| 35 | (end of life adj2 care).tw. |
| 36 | (short-term integrated palliative and supportive care).mp. [mp=title, abstract, original title, name of substance word, subject heading word, floating sub-heading word, keyword heading word, organism supplementary concept word, protocol supplementary concept word, rare disease supplementary concept word, unique identifier, synonyms] |
| 37 | 29 or 30 or 31 or 32 or 33 or 34 or 35 or 36 |
| 38 | ((place or location or site) adj2 (death or dying or die or mortal*)).mp. [mp=title, abstract, original title, name of substance word, subject heading word, floating sub-heading word, keyword heading word, organism supplementary concept word, protocol supplementary concept word, rare disease supplementary concept word, unique identifier, synonyms] |
| 39 | ((home or house or hospital or long-term care facilit* or assisted living) adj2 (death or dying or die or mortal*)).tw. |
| 40 | 38 or 39 |
| 41 | 4 and 16 and 23 |
| 42 | 4 and 16 and 28 |
| 43 | 4 and 16 and 37 |
| 44 | 4 and 16 and 40 |
| 45 | 41 or 42 or 43 or 44 |
